# Adult Hippocampal Neurogenesis and Alzheimer's Disease: Novel Application of Mesenchymal Stem Cells and their Role in Hippocampal Neurogenesis

**DOI:** 10.22088/IJMCM.BUMS.10.1.1

**Published:** 2021-05-22

**Authors:** Mahdi Noureddini, Saeid Bagheri-Mohammadi

**Affiliations:** 1 *Department of Physiology, Faculty of Medicine, Kashan University of Medical Sciences, Kashan, Iran.*; 2 *Department of Applied Cell Sciences, Faculty of Medicine, Kashan University of Medical Sciences, Kashan, Iran.*; 3 *Department of Physiology and Neurophysiology Research Center, School of Medicine, Shahid Beheshti University of Medical Sciences, Tehran, Iran.*

**Keywords:** Alzheimer's disease, cell therapy, hippocampus, neurogenesis, neural stem cells

## Abstract

The neurogenesis can occur in two regions of the adult mammalian brain throughout the lifespan: the subgranular zone of the hippocampal dentate gyrus, and the subventricular zone of the lateral ventricle. The proliferation and maturation of neural progenitor cells are tightly regulated through intrinsic and extrinsic factors. The integration of maturated cells into the circuitry of the adult hippocampus emphasizes the importance of adult hippocampal neurogenesis in learning and memory. There is a large body of evidence demonstrating that alteration in the neurogenesis process in the adult hippocampus results in an early event in the course of Alzheimer's disease (AD). In AD condition, the number and maturation of neurons declines progressively in the hippocampus. Innovative therapies are required to modulate brain homeostasis. Mesenchymal stem cells (MSCs) hold an immense potential to regulate the neurogenesis process, and are currently tested in some brain-related disorders, such as AD. Therefore, the aim of this review is to discuss the use of MSCs to regulate endogenous adult neurogenesis and their significant impact on future strategies for the treatment of AD.

Alzheimer's disease (AD) is a progressive and neurodegenerative disorder of the hippoca-mpus, and is characterized by early neuronal loss. In AD brains, two pathological characteristics are showed including extracellular senile plaques (insoluble) formed by amyloid-β (Aβ) peptide, and intracellular neurofibrillary tangles formed by tau protein (1). Typically, the symptoms of AD begin with mild memory difficulties and evolve towards cognitive impairment, dysfunctions in complex daily activities, and several other aspects of cognition (2). Unfortunately, despite the huge public health problem that it poses, only some medical treatments that control symptoms of the disease have been approved for AD (3), but there is no effective treatment to prevent the neurodegenerative process (Table 1). One important implication for future therapeutic approaches to AD treatment is the heterogeneity of neuropathological conditions. This is of particular concern since the proportion of elderly with undetected neurodege-nerative disease is expected to increase with the age of the population, which may result in invalid conclusions of accelerated age-related decline in cortical regions sensitive to disease pathology, especially the hippocampus (4). Studies on the changes of hippocampal structures reveal that alterations in myelination, neuronal plasticity, and interneuronal connectivity are important for disease development (5). Correspondingly, studies reported that mutations of amyloid precursor protein (*APP*) and presenilin (*PSEN*) genes result in higher brain levels of inflammatory mediators including interleukin (IL)-1β along with the accumulation of autophagic vesicles within dystrophic neurons in the hippocampus (6). In AD conditions, neuronal loss within the hippocampal formation and an extended neuronal network involving the medial temporal and medial parietal lobe can lead to archetypal memory impairment (7, 8). Cumulative evidence has demonstrated that Aβ oligomers have a critical role in neuronal dysfunction and AD pathogenesis (9). Also, many types of research have shown that stem cell therapy can reduce the toxicity and aggregation of Aβ peptides in the hippocampal formation in AD models, as well as promote neurogenesis, and prevent hippocampal damage(7-11).

In the past years, researches showed that the adult brain of mammals has the capacity to produce new cells from neural stem/progenitor cells (NSCs/ NPCs) (12). New investigations demonstrated plasticity-related changes in brain areas with neurogenesis such as the subventricular zone (SVZ) and hippocampal formation in AD. Considering the role of stem cells for brain repair and their role in hippocampal functions such as learning, cognition, and memory, the relevant molecular mechanisms for NSCs and AD etiology should be investigated. Therefore, more information regarding molecular regulation of adult NSCs will improve our insight into the role of adult neurogenesis in the process of degeneration and maybe regeneration in the AD brain (13, 14). 

**Table 1:** Summary of currently available therapies for Alzheimer's disease (AD).

**Treatments**	**Target of AD**	**Results**
Melatonin treatment (Experimental)	Inhibits Aβ aggregation; Restores mitochondria function and lessens the load of oxidative stress	Greatly helps sleep disorders and slows cognitive impairments
Young lymphocyte infusion (Experimental)	Changes the molecular, structure, and cognitive function of older animals	Reverses the effects of aging in the brain of animals
Caffeine treatment (Experimental)	Blocks β- secretases and γ- secretases forming Aβ monomers	Helps reducing cognitive impairment in the animal
Free radical scavengers and anti-inflammatory drugs (Experimental)	Oxidative stress and free radicals in the AD brain	Some improvement in terms of Aβ deposition, inflammation, and symptoms
NMDA receptor antagonist (FDA approved)	Regulates glutamate activity and NMDA receptor activity; lessens excess glutamate and calcium into neuronal cells	Slows down cognitive symptoms progression but not disease progression
Cholinesterase inhibitors (FDA approved)	Prevent breakdown of acetyl choline in the brain; important for learning and memory	Slow down cognitive symptoms progression but not disease progression

In the present paper, we highlight the relations between adult neurogenesis and AD, and try to show the modulation of neurogenesis in the hippocampal formation.

## Adult hippocampal neurogenesis

Adult hippocampal neurogenesis by generating new neurons in the cerebral tissue throughout life represents a strong fascination for scientists and the public alike (15, 16). Based on studies, the adult hippocampus can produce new neurons during lifespan and depending on cellular contexts (17, 18). One of the stem-cell-containing niches in the adult mammalian brain is the subgranular zone (SGZ) of the hippocampal dentate gyrus (DG) (19, 20). Several multistep models have been proposed to schematize the neurogenic cascade. Four distinct phases have been described in the neurogenesis process: phase 1) precursor cells; phase 2) early survival stage; phase 3) postmitotic maturation, and phase 4) late survival stage (21).

**Fig. 1:**
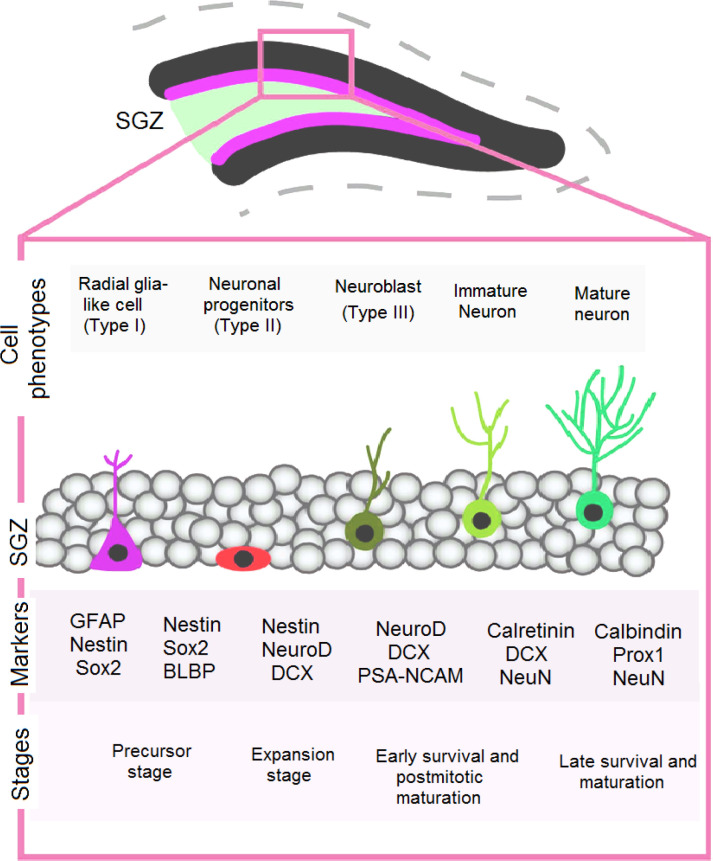
Schematic representation of the anatomical organization of the dentate gyrus, which includes the subgranular zone (SGZ). Neural stem cells (NSCs) are found within the SGZ. Neurogenesis in the dentate gyrus begins with Type I cells entering the cell cycle and proliferating, producing Type II cells and then neuroblasts (Type III cells). These neuroblasts develop into immature neurons (Type IV cells), which ultimately give rise to mature granule cells (Type V cells). Different cellular populations within the dentate gyrus can be identified via the expression of proteins including SOX2, GFAP, DCX, Ki67, TBR2, NeuN, etc

For adult NSCs, there is a thin strip between the hilus and granule cell layer which is considered as a unique microenvironment (22). During the evaluation of various phases of neuronal maturation in the human brain (DG), consideration of phylogenetic differences between humans and other mammals is essential (23). Giving an example, only in humans, striatal neurogenesis is seen (16), but neurogenesis is absent in the olfactory bulb of human beings (24). As shown by Gonçalves *et al*. (2016), the neo-neuron generation in DG neurogenic niche is initiated from quiescent radial-glia-like type I neural progenitor cells (QNPs). These cells can express sex-determining region Y-box 2 (*SOX2*), nestin, brain lipid-binding protein (*BLBP*), and glial fibrillary acid protein (*GFAP*) (23). The asymmetric division of QNPs results in the generation of type II, or amplifying intermediate neural progenitors expressing nestin and Ki-67 (24). SOX2 was also able to reprogram pericytes in the brain into induced neurons, suggesting that its effects were not unique to astrocytes. Reprogram-ming of endogenous astrocytes to neuroblasts with proliferating capacity was observed by delivering neural transcription factor Sox2 into adult mouse striatum. Cortical glial cells rendered reactive by stab wound injury or AD pathology were reprogrammed by neurogenic differentiation 1 (NeuroD1) to form glutamatergic neurons and GABAergic neurons (25). Based on Semënov (2019) study, the number of QNPs decreased about 100 fold from the age of three weeks to the age of 24 months, confirming that QNPs have this characteristic of neural transit cells. Stem cells are responsible for the maintenance of the transit cell population. Thus, reduced QNPs numbers imply that SGZ possesses no active NSCs capable to replace used up QNPs (26). In recent years, it has been speculated that substantial amounts of hippocampal neurogenesis (about seven hundred new cells per day) takes place in DG of the human brain (Figure 1) (27). The human striatum, which contains a neural progenitor population and a dense vascular network linked with NSCs, has been suggested as a source of adult neurogenesis (22).

Impaired adult neurogenesis has been observed in association with various neurodegenerative diseases such as AD. Neurogenesis plays an important functional significance for the treatment of cognitive disorders (28, 29). Multipotent and self-renewing NSCs are responsible for ongoing neurogenesis in the adult hippocampus. During the neurogenesis process, adult NSCs change regarding their intrinsic properties such as gene expression, morphology and self-renewal capacity, and proliferation kinetics. In a normal brain, neurogenesis and integration of new neurons into preexisting neural circuits exert a vital role in hippocampus-dependent learning and memory. However, neurogenesis is affected by injury-associated signals in pathological conditions due to changes in proliferation of NSCs and altered survival and fate of newborn cells (30, 31). According to the investigation in AD patients, modifications in main signals such as PSEN1, Notch1, APP (soluble form), cAMP response element-binding protein (CREB), and β-catenin can regulate disturbed neurogenesis (32). The soluble form of APP can regulate the proliferation and survival of NPCs (32, 33). Notch1 as a critical neurogenic signal and a substrate of γ-secretase (33), CREB as a critical signaling factor for neuronal plasticity and learning (21), and Wnt/β-catenin as critical signaling factors in the regulation of hippocampal neurogenesis can modulate brain plasticity. In the SGZ of the DG, *WNT3* can be expressed, and its overexpression results in increasing neurogenesis. Astrocytes can produce WNTs in the adult hippocampal niche, and support the proliferation and differentiation of NPCs (29). In the adult brain tissue, PSENs are essential for progenitor cell maintenance, as embryonic PSEN deletion results in depletion of stem cells as well as premature neuronal differentiation. Given the important role of PSEN1 in the development of cerebral tissue, the ablation of a cell-intrinsic role of PSEN1 on the adult neurogenesis was unexpected. Verily, embryonic ablation of PSEN1 results in a significant decrease in the number of the NSCs due to the early exit from the cell cycle and premature differentiation into adult cells which are attributed to a blockade of the Notch pathway. The NOTCH is required for the maintenance of embryonic progenitor cells, and is a substrate of γ-secretase, of which PSEN is the catalytic subunit. In adult NSCs, functional analysis of Notch1 signaling and the Notch-pathway genes have shown that a lack of NOTCH1 or RBPJκ (downstream transcriptional effectors) result in suppression of neurogenesis process by depleting the NSCs pool, similar to its effects in the embryo in the hippocampal formation (34, 35).

Based on Tobin *et al*. (2019) study, here we can mention some advantages of endogenous adult hippocampal neurogenesis (18). First, it was revealed that the hippocampal neurogenesis can occur in many aged human brains. So, adult hippocampal neurogenesis can provide critical insight into the role of neurogenesis in human cognition and hippocampal function. Second, scientists showed that better cognitive ability may be leading to higher neurogenesis in the brain. Therefore, adult hippocampal neurogenesis is an active process in the adult mammalian brain, including the human brain (18). In contrast, the number of neurons and their maturation in the hippocampus was sharply reduced as AD advanced. These results show the persistence of adult cerebral neurogenesis during aging in humans and introduce documents for disturbed neurogenesis as a potentially relevant mechanism underlying learning and memory deficits in AD, and may represent effective therapeutic approaches (36).

## Adult hippocampal neurogenesis in Alzheimer's disease

The neurogenesis in hippocampal formation plays a critical role in neuronal plasticity and network maintenance (37). The hippocampal formations as critical regions for learning and memory are especially sensitive in the primary stages of AD. It has been approved in the adult hippocampus that altered neurogenesis can represent an early essential event in the course of AD. Based on studies, it has been demonstrated that different key factors are involved in AD pathogenesis that may impact the generation of new neurons (37). For instance, the proliferation of NSCs/NPCs in the adult brain can be affected by growth factors, including epidermal growth factor (EGF), fibroblast growth factor (FGF) 2, brain-derived neurotrophic factor (BDNF), stem cell factor, heparin-binding EGF (HB-EGF), and vascular endothelial growth factor (VEGF). Also, restoring insulin-like growth factor (IGF) I levels enhances neurogenesis in the aged brain, showing that neurogenesis may be increased by growth factors *in vivo *in neurological disorders like AD (38). Furthermore, some factors central to AD have been shown to play a modulatory role in adult neurogenesis. Experimentally, using a loss of function animal model by crossing apolipoprotein E (*Apo-E*) deficient mice to a nestin-GFP reporter revealed that ablation of *Apo-E* improves the proliferation of early NPCs within the hippocampal formation (DG), which leads to a decrease in the number of Type 1 NPCs over time (39, 40). An age-dependent reduce in SGZ proliferation was also occurred in animals transgenic for human V717F mutant APP (a model of AD). Furthermore, adult neurogenesis was reduced in mice with a knock-out for *Apo-E* or with knock-in alleles for human *APO-E4*. The activation of bone morphogenetic protein (BMP) pathways in *Apo-E*-knock-out animals resulted in enhancement of glial cell differentiation at the expense of neurogenesis, whereas presynaptic GABAergic input mediated the maturation of newborn cells which were diminished in *APO-E4*-knock-in animals because of decreased interneuron survival. Potentiating GABAergic signaling restored neuronal maturation and neurogenesis in *APO-E4*-knock-in mice to normal levels (41, 42). Despite a large amount of data generated from researches employing animal models of AD, how neurogenesis in the hippocampus responds to AD in humans is yet undetected (43).

The disturbance of neurogenesis can be leading to early subtle disease manifestations which results in memory impairment, whereas increase of neurogenesis may introduce an endogenous cerebral repair mechanism. Considering increased adult hippocampal neurogenesis and gene expression changes, stem cell therapy may offer a novel approach to modulate hippocampal activity. Thus, the development of adult neurogenesis cell-based therapies in the human brain might open new horizons for therapeutic strategies in neurodege-nerative diseases as AD.

## Modulation of adult hippocampal neurogenesis

**Fig. 2:**
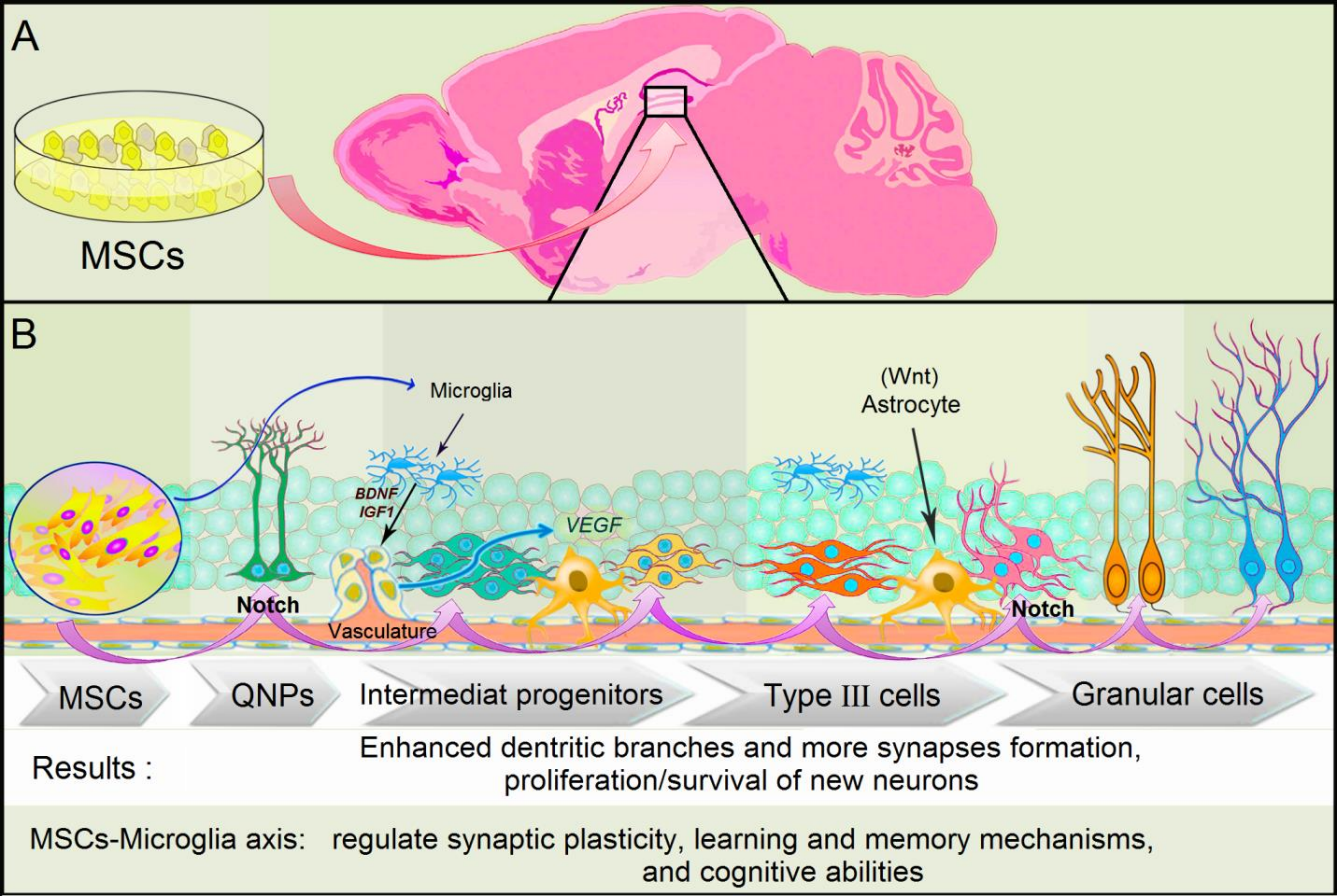
**The hippocampal neurogenesis in the adult brain.** A) The subgranular zone (SGZ) of the hippocampal dentate gyrus (DG) niche in the rodent brain received mesenchymal stem cells; B) Adult MSCs can naturally secrete trophic factors and cytokines which are important for the generation and differentiation of newborn neurons in neurogenic regions of the adult brain such as SGZ of the dentate gyrus. Quiescent radial-glia-like type I neural progenitor cells (QNPs) can generate proliferating intermediate progenitor cells with transient amplifying characteristics. These intermediate progenitor cells can give rise to type III cells and subsequently differentiate into dentate granule neurons such as immature and mature granule neurons. The Notch signaling pathway has a critical role in maintaining active NSCs in the developing CNS. Moreover, *WNT3* is expressed in the SGZ of the DG, and its overexpression is essential to increase neurogenesis. WNTs can be produced by astrocytes in the adult hippocampal niche, and support the proliferation and differentiation of neural progenitor cells. Besides stem cell factors can modulate microglial functions and induce activation of the neuroprotective effects of microglia. Brain-derived neurotrophic factor (BDNF) produced by microglia can modulate motor learning-dependent synapse formation. Furthermore, vascular endothelial growth factor (VEGF) can be up-regulated in AD-MSCs-treated animals. VEGF is a signal protein produced by cells that regulate vascular permeability and angiogenesis

Today, stem cell-based therapy using mesenchymal stem cells (MSCs) has emerged as a promising strategy for modulation of the adult hippocampal niche. MSCs could pass the blood-brain-barrier by migration with the aid of concomitantly infused stimulators (35, 44). Transplanted immunomodulatory MSCs have shown to generate and differentiate newborn neurons in neurogenesis in SVZ and hippocampal regions and booster memory functions through the secretion of cytokines and trophic factors important for pro and anti-inflammatory modulation (45-48). MSCs improve cognition ability through the restoration of damaged synaptic circuits in the hippocampal formation and activation of some signaling axis including BDNF-TrkB-CREB (49-51). Adipose tissue is considered a rich source of MSCs, up to 500 times compared with bone marrow (49, 52, 53), containing an abundant vascularization. In 2018, Hyo Rim Ko *et al*. showed that treatment with MSCs reduced neuronal loss and improved neurogenesis in the hippocampal formation (Figure 2). NSCs are localized in SVZ or SGZ of the hippocampus, which is quiescent and could proliferate under-stimulation to become neurons or glial cells after migration. This can imply the link between impaired SVZ and / or DG neurogenesis seen in AD (51, 54). MSCs are known for their ability to promote the neurogenesis of primary NPCs and survival of neural cells by expressing neurotrophic factors, such as BDNF, β-nerve growth factor (NGF), and IGF-1. Moreover, the application of MSCs in the cerebral tissue prevents apoptosis and improves neurogenesis (proliferation and differentiation) in the engrafted area (55). According to a study, engraftment of MSCs into the hippocampus and dentate gyrus of Flinders Sensitive Line animals resulted in enhanced neurogenesis and affected behavioral changes, reversing the depressive-like phenotype normally presented by these animals (55).

In neurological diseases, the possibility of employing NSCs to re-cover specific regions of the central nervous system (CNS) is highly appealing. Based on studies, calcium signaling homeostasis balance is also important for the NSCs fate phenotypic choice, highlighting the importance of the mechanism that controls the pool size of NSCs in the adult brain. Calcium transient is involved in both astrocytogenesis and neuronal fate choice. Astrocytes also trigger neurogenic response and behavior by activation of NPCs through intracellular calcium concentration transients (29, 56, 57). Many neurological diseases such as AD show that calcium misbalance could initiate an apoptotic response with subsequent neurodeg-eneration and massive neuronal loss. Newly, some studies revealed the state's deregulation of intracellular calcium concentration homeostasis as the disease cause. Many previous cell-transplantation showed that stem cells as a strong tool to modulate calcium signaling in neurological diseases can be considered as an effective therapeutic strategy for the treatment of AD (57-59). MSCs were therefore suggested as candidates for treating a variety of neurodegenerative diseases; in particular, they can modulate brain homeostasis. It can be noted that alteration in extrinsic factors during the adult hippocampal neurogenesis process, including environmental conditions, diet compo- sitions, and regulatory factors of metabolism are also known to regulate adult hippocampal neurogenesis (60, 61). For example, physical exercise has been demonstrated to improve metabolism enhanced adult hippocampal neurogenesis, hippocampus-related cognitive function, and synaptic plasticity (62-65). Therefore, a combination of stem cell therapy and other therapeutic strategies may offer a new approach to regulate the hippocampal activity.

## Challenges and future directions for stem cell technology

Stem cell technology has brought a promising translational significance as could be seen by data regarding their therapeutic benefits in AD, but still; several challenges have remained in its development. Items including type, sources, stages, doses, and transmission routes should be validated, but considering donor-to-donor variation and genetic and epigenetic backgrounds of donor cells is essential. Although the effectiveness of stem cell therapy in AD has not yet been verified, its appliance for AD disease treatment is expected. Stem cell technology for treating AD is a promising way of modifying the disease pathology, but because that is still in infancy, stem cell therapy in AD is becoming more widespread and sophisticated.

In conclusion, our review shows the potent effect of stem cells on brain homeostasis, and suggests that further development of cell-based therapy or other strategies that modulate hippocampal neurogenesis can provide a viable approach to the treatment of AD.
